# High expression levels of influenza virus receptors in airway of the HBV-transgenic mice

**DOI:** 10.1017/S0950268819001833

**Published:** 2019-11-04

**Authors:** Jiajun Yang, Hao Li, Liyuan Jia, Xianchun Lan, Yuhui Zhao, Huijie Bian, Zheng Li

**Affiliations:** 1 Laboratory for Functional Glycomics, College of Life Sciences, Northwest University, Xi'an 710069, China; 2 Cell Engineering Research Centre and Department of Cell Biology, Fourth Military Medical University, Xi'an 710032, China; 3 College of Medicine, Xi'an International University, Xi'an 710077, China

**Keywords:** *α*2-3/6-linked sialic acids, airway, influenza A virus, liver disease, receptors

## Abstract

In the human population, influenza A viruses are associated with acute respiratory illness and are responsible for millions of deaths annually. Avian and human influenza viruses typically have a different *α*2-3- and *α*2-6-linked sialic acid (SA) binding preference. Only a few amino acid changes in the haemagglutinin on the surface of avian influenza viruses (AIV) can cause a switch from avian to human receptor specificity, and the individuals with pathognostic chronic diseases might be more susceptible to AIV due to the decreased expression level of terminal *α*2-3-linked SA in their saliva. Here, using lectin and virus histochemical staining, we observed the higher expression levels of *α*2-3/6-linked SA influenza virus receptors in the airway of HBV-transgenic mice compared with that of control mice due to the significant decrease in control mice during ageing, which imply that this is also a risk factor for individuals with pathognostic chronic diseases susceptible to influenza viruses. Our findings will help understand the impact on influenza virus pathogenesis and transmission.

## Introduction

In the human population, influenza A viruses (IAV) are associated with acute respiratory illness and are responsible for millions of deaths annually. IAV express two membrane-bound surface glycoproteins, the haemagglutinin (HA) and the neuraminidase (NA) and entry of avian and human influenza viruses (HuIV) is mediated by HA that binds to specific sialyloligosaccharides [1, 2]. Avian influenza viruses (AIV) are considered to be key contributors to the emergence of human influenza pandemics, and its HA preferentially recognises sialic acid (SA) *α*2-3galactose (Gal)-linked receptors, whereas HuIV HA preferentially recognises SA *α*2-6Gal-linked receptors [3–8]. It has been shown that the difference in receptor use between AIV and HuIV, combined with the distribution of their receptors in the respiratory tract, results in a different localisation of virus attachment [9–12]. HuIV attaches more abundantly to the upper respiratory tract and trachea, whereas AIV predominantly attaches to the lower respiratory tract [13–15].

The oral cavity can be an entry site of the influenza virus, mucus present in the respiratory tract from the human respiratory tract predominately contains *α*2-3/6-linked SA receptors, which may hamper influenza virus binding and infection [16, 17]. Moreover, saliva is known to be a potentially important barrier against IAV infection. The barrier action by saliva can be explained by two distinct mechanisms [18]. One mechanism involves proteins (pulmonary surfactant protein D) binding via carbohydrate-recognition domains to carbohydrates on the HA1 domain of the HA and on the NA of IAV [19–21]. The other mechanism involves proteins (*α*-2-macroglobulin, MUC5B and salivary glycoprotein-340) inhibiting IAV by presenting a SA ligand for viral HA [22–25]. Our previous study indicated that elderly individuals had stronger resistance to IAV partly by presenting more terminal *α*2-3/6-linked SA residues in their saliva to bind with viral HA and inhibit the activities of IAV [26]. However, it is often noted that hospitalisations and deaths after an influenza infection mainly occur in the elderly population diagnosed with chronic diseases, such as diabetes and cancer [27–30]. This phenomenon is usually explained by the fact that chronic diseases reduce the ability of the immune system to fight infections. Our further study provided evidence that elderly individuals with chronic diseases, such as diabetes and liver disease, might be more susceptible to AIV due to the decreased expression of terminal *α*2-3-linked SA residues in their saliva [31].

In the current study, we employed the HBV-transgenic mice C57BL/6J-TG(ALB1HBV)44BRI/J that develop progressive hepatic damage, which well mimics the natural history of human hepatocarcinogenesis, and assessed the *in situ* tissue expression levels of both *α*2-3/6-linked SA influenza virus receptors in the respiratory tract of HBV-transgenic mice. Then, we further evaluated their binding capacities with one strain (A/Duck/Guangdong/17/2008 (H5N1)) of AIV and H1N1 influenza A vaccine. The findings from this study will also help unravel the molecular mechanism of patients with chronic diseases susceptible to IAV and find effective prevention and treatment agents against IAV.

## Materials and methods

### Animal

HBV-transgenic mice C57BL/6J-TG(ALB1HBV)44BRI/J which contain HBV genome S, pre-S and X domains under the mice albumin promoter and can develop progressive hepatic damage that well mimics the natural history of human hepatocarcinogenesis, were provided by The Jackson Laboratory (Bar Harbor, ME) [32]. The negative control of C57BL/6 mice was purchased from VITALRIVER experiment animal company (Beijing, China). The HBV-transgenic mouse and C57BL/6 mouse colonies were maintained in the Cell Engineering Research Centre and Department of Cell Biology of Fourth Military Medical University (Xi'an, China). Ethical approval for the current study was obtained from The Laboratory Animal Ethics Committee of Northwest University and Fourth Military Medical University. The current study was performed in strict accordance with the People's Republic of China Legislation Regarding the Use and Care of Laboratory Animals.

### Formalin-fixed, paraffin-embedded tissue

Fifteen, 10 to 18 month old HBV-transgenic mice (*n* = 3, at the same age) and 15, 10 to 18 month old C57BL/6 mice (*n* = 3, at the same age) were anaesthetised and sacrificed for tissue material sampling. Fresh trachea and lung of mice were fixed in 4% paraformaldehyde for 24 h, then rinsed in running water for 12 h. Then, they were dehydrated in two changes of 70% ethanol for 30 min each, followed by 80%, 90%, 95%, 95%, 100% and 100% ethanol for 20 min each. They were transparentised in a mixture of xylene with ethanol (1:1), followed by two changes of xylene for 30 min each; and embedded in three changes of paraffin wax for 30 min each. All of the paraffin-embedded tissues were cut into 4 µm thick, and attached to the glass slides.

### Dewaxed and rehydrated

Formalin-fixed, paraffin-embedded tissue sections were dried overnight at 60 °C. Paraffin was removed from sections by immersing slides in two changes of xylene, 5 min each; and hydrated in two changes of 100% ethanol for 5 min each, followed by 95%, 90%, 80% and 70% ethanol for 5 min each, rinsed in running water for 3 min. These dewaxed and rehydrated sections were subjected to the following method.

### Preparation of avian influenza virus

One H5N1 subtype strain (A/Duck/Guangdong/17/2008) was cultured in the chorioallantoic fluid of 10-day-old embryonated hen eggs and then purified on a discontinuous sucrose-density gradient as described previously [33].

### Labelling of lectins and viruses

Maackia amurensis lectin II (MAL-II) and sambucus nigra agglutinin (SNA) (Vector, Burlingame, CA) were labelled with Cy5 fluorescent dye and Cy3 fluorescent dye (GE Healthcare, Buckinghamshire, UK), respectively, and the H5N1 subtype strain and H1N1 vaccine were labelled with Cy5 fluorescent dye [34]. All labelled lectins and viruses were purified using Sephadex G-25 columns, as described previously [35]. Subsequently, the Cy3- and Cy5-labelled lectins and Cy5-labelled viruses were quantified and stored at −20 °C in dark until use.

### Fluorescence-based lectin histochemistry

All the paraffin-embedded sections were stained with fluorescently labelled SA-specific lectins. Cy5-labelled MAL-II, specific for *α*-2,3-linked SA to detect AIV receptors, and Cy3-labelled SNA, specific for the *α*-2,6-linked SA to detect HuIV receptors. The fluorescence-based lectin histochemistry was performed as previously described [33]. Briefly, dewaxed and rehydrated tissue sections were microwaved in 10 mM citrate buffer (pH 6.0) at 100 °C for 10 min and cooled at room temperature to eliminate endogenous peroxidase activity. After rinsed with PBS three times, 5% bovine serum albumin and 0.02% Triton X-100 was used to block nonspecific staining at 25 °C for 1 h. The tissue sections were incubated with a mixed solution containing a final concentration of 100 µg/ml of Cy3-labelled SNA and Cy5-labelled MAL-II each at room temperature for 3 h in dark, respectively. Finally, sections were stained with 1 µg/ml of DAPI (Roche, Basel, CH) for 10 min before the final rinse.

### Fluorescence-based virus histochemistry

All the paraffin-embedded sections were bound with fluorescently labelled viruses. The steps were similar to fluorescence-based lectin histochemistry. After blocked, the tissue sections were incubated with 100 µg/ml of Cy5-labelled H5N1 subtype strain and H1N1 vaccine at 4 °C overnight in dark, respectively. Finally, sections were stained with 1 µg/ml of DAPI (Roche, Basel, CH) for 10 min before the final rinse.

### Statistical analysis

Three independent biological replicates were performed. A laser scanning confocal microscope FV 1000 (Olympus, Tokyo, Japan) was used to collect the images using the merge channels of Cy5, Cy3 and DAPI and analysed with ImageJ and SPSS software. To evaluate the expression levels of influenza virus receptors, random area of every tissue section was chosen to capture and the average fluorescent intensity (AFI) was calculated. The AFI of three independent biological replicates in tissue pairs were compared with each other based upon fold-changes, according to the following criteria: fold change >1.5 or <0.67 in tissue pairs indicated up-regulation or down-regulation, and *P*-values lower than 0.05 were considered statistically significant.

## Results

### Expression levels of *α*2-3/6-linked SA receptors in the trachea of HBV-transgenic mice

In order to determine the expression levels of *α*2-3/6-linked SA receptors in the trachea of the HBV-transgenic mice model compared with control mice, the tissue sections of trachea from 10 to 18 month old mice were stained with Cy5-labelled MAL-II and Cy3-labelled SNA, respectively. Besides, the labelled Cy5/Cy3-BSA staining was used as negative controls. Specifically, random area of every tissue section was selected to capture and the AFI was calculated. The fold-changes of the AFI of three independent biological replicates in tissue pairs were calculated, which were classified into three categories to evaluate whether the expression levels of *α*2-3/6-linked SA receptors were altered in the respiratory tract of HBV-transgenic mice compared with control mice: (1) results showing significant increases in AFI (fold change ⩾1.50, *P* < 0.05), (2) results showing significant decreases in AFI (fold change ⩽0.67, *P* < 0.05) and (3) results showing an almost even level in AFI (fold change range from 0.67 to 1.50, no significant difference).

**Fig. 1.**
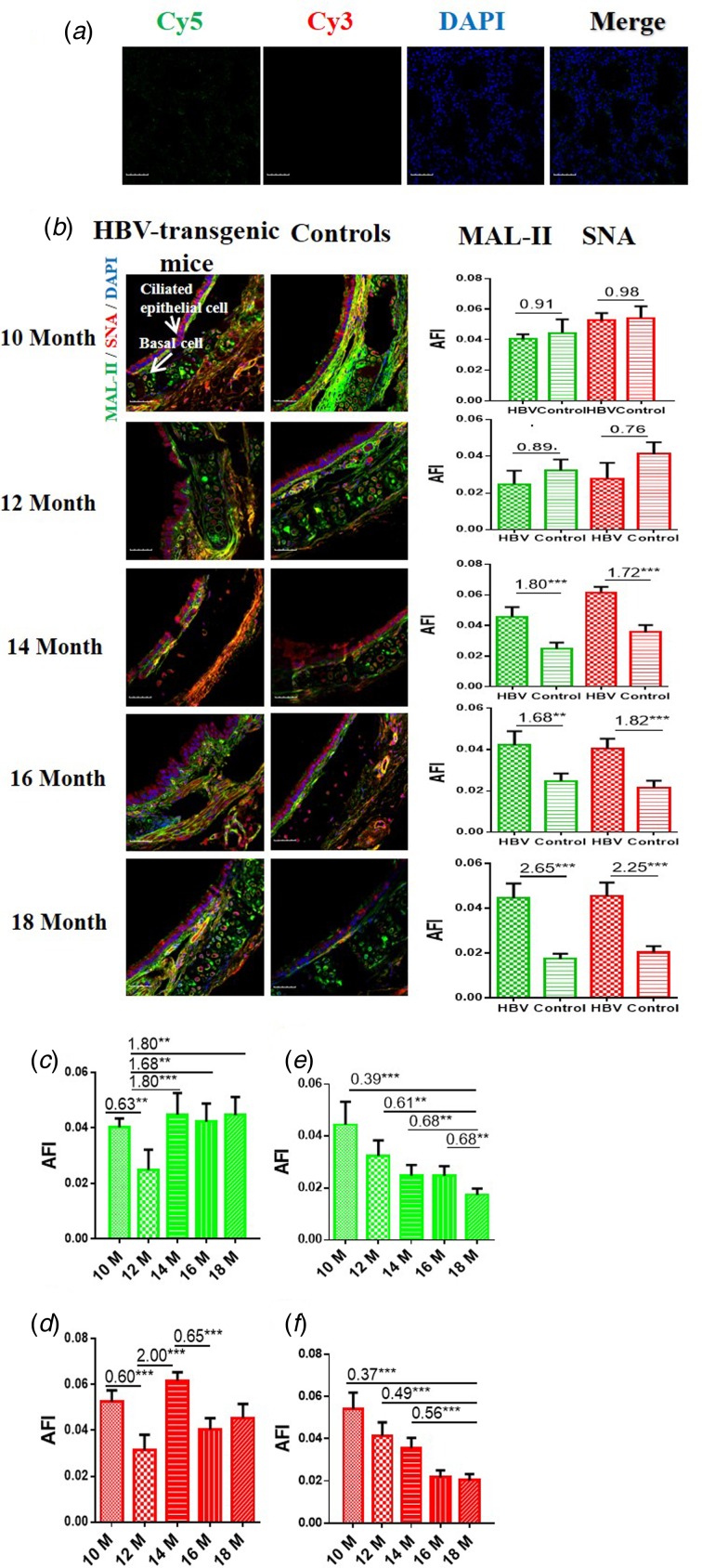
(a) Cy5 and Cy3-labelled BSA staining were used as negative controls. (b) Expression levels of *α*2-3/6-linked SA receptors in the trachea tissues of HBV-transgenic mice and control mice at different ages. (c, d) The expression levels of *α*2-3/6-linked SA receptors in the trachea tissues of HBV-transgenic mice with ageing. (e, f) The expression levels of *α*2-3/6-linked SA receptors in the trachea tissues of control mice with ageing. Green bar: Cy5-labelled MAL-II, specific for *α*2-3-linked SA to detect AIV receptors. Red bar: Cy3-labelled SNA, specific for the *α*2-6-linked SA to detect HuIV receptors. The images of tissue pairs were acquired using the merge channels of Cy5, Cy3 and DAPI. Scale bar = 50 µm.

The results showed that the negative controls had no positive signal (Fig. 1a) and both HBV-transgenic mice and control mice expressed *α*2-3/6-linked SA receptors on the ciliated epithelial cells and basal cells of the trachea (Fig. 1b). The expression levels of *α*2-3/6-linked SA receptors showed an almost even level in the trachea tissues of 10 and 12 month old HBV-transgenic mice compared with control mice, however, they were significantly higher in the trachea tissues from 14 to 18 month old HBV-transgenic mice than that of control mice (all fold change ⩾1.68, *P* < 0.01). Notably, the expression levels of *α*2-3/6-linked SA receptors had no significant difference in the trachea of the HBV-transgenic mice model accompanying by progressive hepatic damage (from 10 to 18 months) (Fig. 1c and d), however, they showed a decreasing trend in the trachea of control mice with ageing (from 10 to 18 months) (Fig. 1e and f).

### Binding of influenza A virus with the trachea of HBV-transgenic mice

The capacities of influenza viruses bound with SA receptors in the trachea of HBV-transgenic mice model and control mice were also assessed. The tissue sections of trachea from 10 to 18 month old mice were incubated with Cy5-labelled one strain (A/Duck/Guangdong/17/2008(H5N1)) and H1N1 vaccine, respectively. Similarly, random area of every tissue section was selected to capture, and then the AFI was calculated. The fold-changes of the AFI of three independent biological replicates in tissue pairs were calculated, which were classified into three categories to evaluate whether the ability of influenza viruses bound with their receptors was altered in the respiratory tract of HBV-transgenic mice compared with control mice: (1) results showing significant increases in AFI (fold change ⩾1.50, *P* < 0.05), (2) results showing significant decreases in AFI (fold change ⩽0.67, *P* < 0.05) and (3) results showing an almost even level in AFI (fold change range from 0.67 to 1.50, no significant difference).

**Fig. 2.**
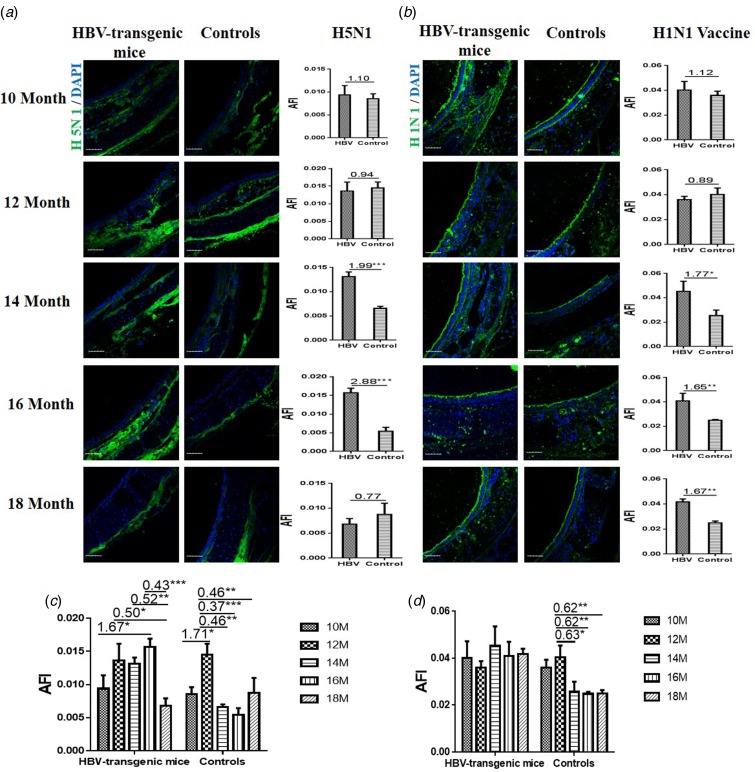
Assessment of binding capacities of IAV with the SA receptors in the trachea tissues of the HBV-transgenic mice and control mice. (a) The binding of H5N1 subtype strain with the trachea tissues at different ages. (b) The binding of H1N1 vaccine with the trachea tissues at different ages. (b) The binding of H5N1 subtype strain with the trachea tissues with ageing. (d) The binding of H1N1 vaccine with the trachea tissues with ageing. The images of tissue pairs were acquired using the merge channels of Cy5 and DAPI. Scale bar = 50 µm.

The results showed that the capacity of H5N1 subtype strain binding to the trachea tissues was an almost even level in 10 and 12 month old HBV-transgenic mice compared with control mice, however, there were more H5N1 viral particles bond to trachea tissues from 14 to 16 month old HBV-transgenic mice compared with control mice (all fold change ⩾1.99, *P* < 0.001) (Fig. 2a). The capacity of HuIV (H1N1 vaccine) binding to the trachea tissues was also an almost even level in 10 and 12 month old HBV-transgenic mice compared with control mice, however, there were more HuIV viral particles bond to trachea tissues from 14 to 18 month old HBV-transgenic mice compared with control mice (all fold change ⩾1.65, *P* < 0.05) (Fig. 2b). Notably, the capacity of H5N1 strain binding to the trachea tissues showed increasing trend in the 10–16 month old HBV-transgenic mice model accompanying by progressive hepatic damage, and then its capacity decreased significantly from 16 to 18 months, however, its capacity showed a decreasing trend in the control mice with ageing (from 12 to 18 months) (Fig. 2c). The capacity of H1N1 vaccine binding to the trachea tissues also had no significant difference in the HBV-transgenic mice model accompanying by progressive hepatic damage, but its capacity showed a decreasing trend in the control mice with ageing (from 10 to 18 months) (Fig. 2d).

### Expression levels of α2-3/6-linked SA receptors in the lung of HBV-transgenic mice

**Fig. 3.**
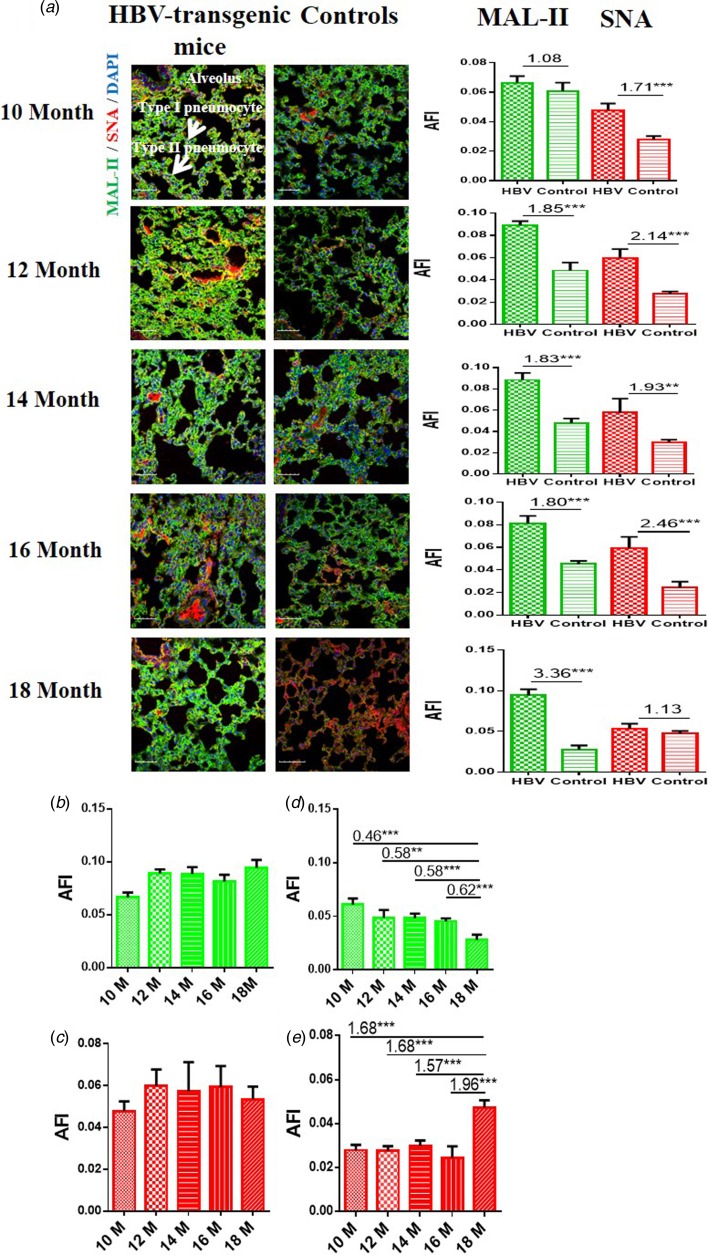
(a) Expression levels of *α*2-3/6-linked SA receptors in the lung tissues of HBV-transgenic mice and control mice at different ages. (b, c) The expression levels of *α*2-3/6-linked SA receptors in the lung tissues of HBV-transgenic mice with ageing. (d, e) The expression levels of *α*2-3/6-linked SA receptors in the lung tissues of control mice with ageing. Green bar: Cy5-labelled MAL-II, specific for *α*2-3-linked SA to detect AIV receptors. Red bar: Cy3-labelled SNA, specific for the *α*2-6-linked SA to detect HuIV receptors. The images of tissue pairs were acquired using the merge channels of Cy5, Cy3 and DAPI. Scale bar = 50 µm.

In order to determine the expression levels of *α*2-3/6-linked SA receptors in the lung of the HBV-transgenic mice model compared with control mice, the tissue sections of lung from 10 to 18 month old mice were stained with Cy5-labelled MAL-II and Cy3-labelled SNA, respectively. Similarly, random area of every tissue section was selected to capture, and then AFI was calculated. The fold-changes of the AFI of three independent biological replicates in tissue pairs were calculated. The results showed that both HBV-transgenic mice and control mice expressed *α*2-3-linked and *α*2-6-linked SA receptors in their pulmonary alveoli (Fig. 3a). The expression level of *α*2-3-linked SA receptor was significantly higher in the lung tissues from 12 to 18 month old HBV-transgenic mice than that of control mice (fold change ⩾1.80, *P* < 0.001). Furthermore, the expression level of *α*2-6-linked SA receptor was significantly higher in the lung tissues from 10 to 16 month old HBV-transgenic mice than that of control mice (fold change ⩾1.71, *P* < 0.01). Interestingly, the expression levels of *α*2-3/6-linked SA receptors had no significant difference in the pulmonary alveoli of the HBV-transgenic mice model accompanying by progressive hepatic damage (from 10 to 18 months) (Fig. 3b and c). However, the expression level of *α*2-3-linked SA receptor showed a decreasing trend in the pulmonary alveoli of control mice with ageing (from 10 to 18 months), and the expression level of *α*2-6-linked SA receptor had significantly increased in the pulmonary alveoli of 18 month old control mice (all fold change ⩾1.57, *P* < 0.001) (Fig. 3d and e).

### Binding of influenza A virus with the lung of HBV-transgenic mice

**Fig. 4.**
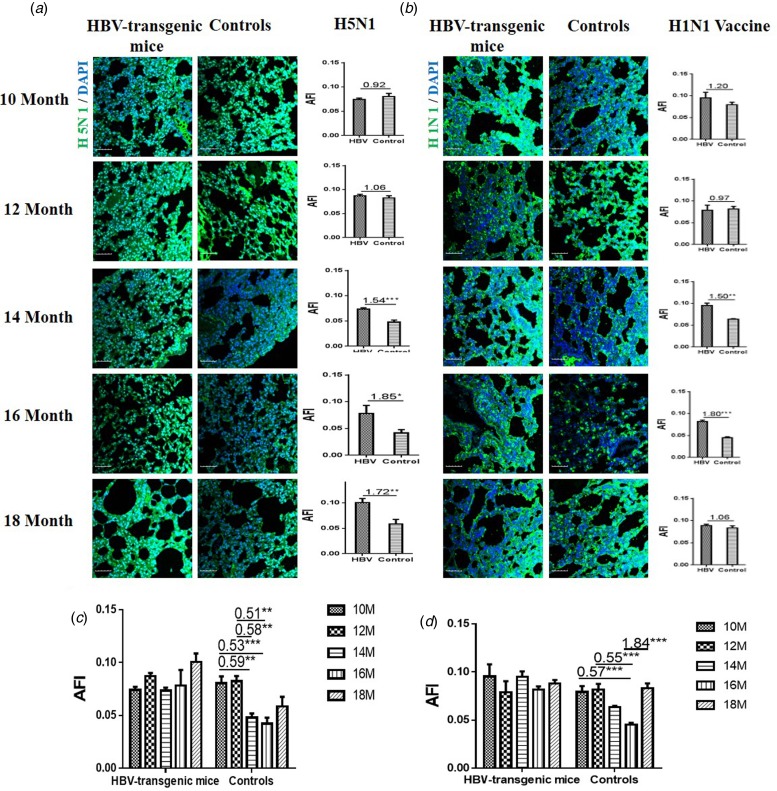
Assessment of binding capacities of IAV with the SA receptors in the lung tissues of the HBV-transgenic mice and control mice. (a) The binding of H5N1 subtype strain with the lung tissues at different ages. (b) The binding of H1N1 vaccine with the lung tissues at different ages. (c) The binding of H5N1 subtype strain with the lung tissues with ageing. (d) The binding of H1N1 vaccine with the lung tissues with ageing. The images of tissue pairs were acquired using the merge channels of Cy5 and DAPI. Scale bar = 50 µm.

The capacities of influenza viruses bound with SA receptors in the lung of HBV-transgenic mice model and control mice were also assessed. The tissue sections of lung from 10 to 18 month old mice were incubated with Cy5-labelled H5N1 subtype strain and H1N1 vaccine, respectively. Similarly, random area of every tissue section was selected to capture and AFI was calculated. The fold-changes of the AFI of three independent biological replicates in tissue pairs were calculated. The capacity of H5N1 strain binding to the lung tissues was an almost even level in 10 and 12 month old HBV-transgenic mice compared with control mice, but there were more H5N1 viral particles bond to lung tissues in 14 to 18 month old HBV-transgenic mice compared with control mice (all fold change ⩾1.54, *P* < 0.05) (Fig. 4a). The capacity of H1N1 vaccine binding to the lung tissues was an almost even level in 10 and 12 month old HBV-transgenic mice compared with control mice, but there were more HuIV viral particles bond to lung tissues from 14 to 16 month old HBV-transgenic mice compared with control mice (all fold change ⩾1.50, *P* < 0.01) (Fig. 4b). Notably, the capacity of H5N1 virus binding to the lung tissues had no significant difference in the HBV-transgenic mice model accompanying by progressive hepatic damage, but its capacity showed a decreasing trend in the control mice with ageing (from 10 to 18 months) (Fig. 4c). The capacity of H1N1 vaccine binding to the lung tissues also had no significant difference in the HBV-transgenic mice model accompanying by progressive hepatic damage, but its capacity showed a decreasing trend in the control mice with ageing (from 10 to 16 months) (Fig. 4d).

## Discussion

Much of the available data on the tropism of influenza viruses in the human respiratory tract has been derived from few autopsy studies of fatal cases. Although highly informative to identify the cell types that are infected *in vivo* and to understand pathogenesis, these data are generally restricted to late phases of the infection [36]. In attempts to compensate for the lack of data in humans, the effect of receptor specificity on tropism, pathogenesis and transmission has also been studied in a number of animal models such as ferrets, pigs, guinea pigs and mice [37, 38]. Our previous study demonstrated that healthy elderly individuals had stronger resistance to IVA partly by presenting more terminal *α*2-3/6-linked SA residues in their saliva to bind with viral HA and inhibit the activities of IVA [26]. However, elderly individuals with chronic diseases, such as diabetes and liver disease (hepatitis B, hepatic cirrhosis, hepatocellular carcinoma), might be more susceptible to AIV due to the decreased expression level of terminal *α*2-3-linked SA in their saliva [31].

In the nasopharynx of humans, *α*2-3/6-linked SA receptors were detected on ciliated cells and mucus-producing cells [35]. In this study, because it is difficult to obtain nasal mucosa in the nasal cavity of HBV-transgenic mice, here, we observed only that the alteration of the expression levels of the *α*2-3/6-linked SA in the tissues of trachea and lung of the HBV-transgenic mice in comparison with that of control mice. In the bronchus, *α*2-3/6-linked SA receptors are heterogeneously distributed in the ciliated and nonciliated cells [39]. The expression levels of *α*2-3/6-linked SA receptors were significantly higher on the ciliated epithelial cells and basal cells of the trachea tissues from 14 to 18 month old HBV-transgenic mice compared with control mice due to a decreasing trend of that in the trachea of control mice with ageing (from 10 to 18 months). Both receptors are abundantly presented in the lower respiratory tract, especially in pulmonary alveoli [40]. Staining of *α*2-3-linked SA receptors using MAA-II was most abundant in cells lining the alveoli, mainly type-II cells [14]. The expression level of *α*2-3-linked SA receptor was significantly higher in the lung tissues of 12 to 18 month old HBV-transgenic mice compared with control mice due to the expression levels of that showed a decreasing trend in the pulmonary alveoli of control mice with ageing (from 10 to 18 months). Furthermore, the expression level of *α*2-6-linked SA receptor was significantly higher in the lung tissues from 10 to 16 month old HBV-transgenic mice compared with control mice due to a decreasing trend in the pulmonary alveoli of control mice with ageing (from 10 to 16 months). The infection of avian and HuIV is mediated by viral HA that binds to specific sialyloligosaccharides on the surface of host cells, after the binding of HA to host SA receptors, the life cycle of IAV is initiated. Then the fusion of viruses and cellular membranes by endocytosis, and viral nucleolar protein complexes are released into the cytoplasm, transported to the nucleus and replicated and transcribed by viral polymerase complexes [40, 41]. Obviously, the alternation of the expression level of SA receptors is closely related to susceptibility and transmission of IAV.

Surface adhesion is achieved by binding HA to the cellular SA receptors. Although virus histochemistry only provides information about viral HA for host cell surface adhesion rather than replication ability of virus, it should be highlighted that viral HA for host cell surface adhesion is a necessary condition in the virus replication cycle [42]. In this study, the capacities of IAV bound with SA receptors in the airway of HBV-transgenic mice model and control mice were assessed, which showed that there were more H5N1 subtype strain virus and H1N1 vaccine binding to the trachea tissues from 14 to 16 or 18 month old HBV-transgenic mice compared with control mice significantly. Similarly, there were more H5N1 subtype strain virus and H1N1 vaccine binding to the lung tissues from 14 to 18/16 month old HBV-transgenic mice compared with control mice significantly. The capacity of H5N1 virus and H1N1 vaccine binding to the airway tissues of mice are almost identical to the distribution of the *α*2-3/6-linked SA receptors. These results implied that individuals with chronic diseases, such as liver disease, might be more susceptible to H5N1 and H1N1 viruses due to the higher expression levels of *α*2-3/6-linked SA influenza virus receptors in their airway in comparison with healthy individuals, and healthy individuals had stronger resistance to AIV due to the decreased expression level of SA receptors in their airway with ageing.

Indeed, chronic diseases, such as chronic pulmonary, heart, kidney, hepatic, neurological diseases and malignancies, are considered to be an important predisposing condition for complicated infection, hospitalisations and deaths from influenza [43–45]. Influenza virus infection may be particularly problematic in children with chronic lung diseases (CLDs), and children with CLDs have been reported to be at higher risk of hospitalisation with influenza [46, 47]. Our results provide an insight into this phenomenon that individuals with chronic diseases, such as liver disease, might be more susceptible to IAV due to the higher expression levels of *α*2-3/6-linked SA influenza virus receptors in their airway in comparison with healthy individuals. However, healthy individuals had stronger resistance to AIV due to the decreased expression level of *α*2-3-linked SA receptor in their airway with ageing.

Our analyses have three limitations. First, we have investigated only the expression levels of *α*2-3/6-linked SA in the airway of the HBV-transgenic mice. Second, our investigation has not referred to the mechanisms that cause the higher expression levels of *α*2-3/6-linked SA in the airway of the HBV-transgenic mice. Another limitation of our study is that anti-influenza activity has been not assessed. In conclusion, AIV and HuIV typically have a different SA-binding preference and only a few amino acid changes in the HA protein can cause a switch from avian to human receptor specificity. The individuals with pathognostic chronic diseases might be more susceptible to AIV due to the decreased expression level of terminal *α*2-3-linked SA in their saliva. Here, we reported the higher expression levels of *α*2-3/6-linked SA influenza virus receptors in the airway of the HBV-transgenic mice in comparison with that of control mice, which is also a risk factor for individuals with chronic diseases susceptible to IAV. Furthermore, the significant difference between the expression levels of SA receptors in the HBV-transgenic mice and the control mice was caused by the decreasing expression in the control mice with ageing. Our findings will help understand the impact on IAV host range, pathogenesis and transmission.
